# Bioinformatics analysis and experimental validation of cuproptosis-related lncRNA LINC02154 in clear cell renal cell carcinoma

**DOI:** 10.1186/s12885-023-10639-2

**Published:** 2023-02-16

**Authors:** Junlin Shen, Linhui Wang, Jianbin Bi

**Affiliations:** Department of Urology, China Medical University, The First Hospital of China Medical University, Shenyang, Liaoning China

**Keywords:** Clear cell renal cell carcinoma, Cuproptosis, Computational biology, Robust model, Risk signature, Immune microenvironment, Drug sensitivity

## Abstract

**Background:**

Clear cell renal cell carcinoma (ccRCC) is common in urinary system tumors. Cuproptosis is a non-apoptotic cell death pathway. Copper binds to fatty acylated mitochondrial proteins and activates various forms of cell death. LncRNA LINC02154 is significantly highly expressed in cells and tissues of many types of tumors, and the risk signature of LINC02154 in some tumors has been validated for effectiveness.

**Methods:**

We constructed a risk prognostic signature by obtaining differentially expressed long noncoding RNAs (lncRNAs) associated with ccRCC outcomes and cuproptosis from The Cancer Genome Atlas (TCGA). We used TCGA to construct training and testing sets to analyze the risk signature and the impact of LINC02154, and we performed relevant survival analyses. Tumor mutational burdens were analyzed in different LINC02154 expression groups and risk score groups. We next analyzed the immune microenvironment of LINC20154. We performed LINC20154-related drug sensitivity analyses. We also investigated the cellular function of LINC02154 in the ACHN cell line and performed CCK-8 assay, EdU, wound-healing assay, and Transwell assay. The essential genes FDX1 and DLST of cuproptosis were detected by western blot.

**Results:**

We demonstrated that LINC02154’s impact on outcomes was statistically significant. We also demonstrated the association of different ages, genders, stages, and grades with LINC02154 and risk models. The results showed a significant difference in tumor mutation burden between the groups, which was closely related to clinical prognosis. We found differences in immune cells among groups with different levels of LINC02154 expression and significant differences in immune function, immunotherapeutic positive markers, and critical steps of the immune cycle. The sensitivity analysis showed that differential expression of LINC02154 discriminated between sensitivity to axitinib, doxorubicin, gemcitabine, pazopanib, sorafenib, sunitinib, and temsirolimus. This difference was also present in the high-risk group and low-risk group. We demonstrated that the proliferation and migration of t ACHN cells in the LINC02154 knockdown group were inhibited. The western blot results showed that the knockdown of LINC02154 significantly affected the expression of FDX1 and DLST, critical genes of cuproptosis.

**Conclusion:**

Finally, we demonstrated that LINC02154 and our constructed risk signature could predict outcomes and have potential clinical value.

**Supplementary Information:**

The online version contains supplementary material available at 10.1186/s12885-023-10639-2.

## Background

Renal cell carcinoma (RCC) includes papillary RCC, clear cell RCC (ccRCC), and chromogenic RCC, with ccRCC accounting for 70% of all kinds of RCC [1]. A high cancer-related death rate is associated with ccRCC, primarily due to metastasis [2]. Surgical treatment is the best means of early treatment [3]. The process of ccRCC is often occult, failing surgical treatment to achieve adequate results [4]. For these reasons, nearly 30% of ccRCC patients have already metastasized at the time of diagnosis, whereas the prognosis of metastatic ccRCC is very poor [5]. There has been a significant improvement in survival with immune checkpoint blockade therapy and combination regimens for patients with ccRCC [6]. Therefore, we hope to identify a biomarker that can help diagnose ccRCC early, provide a basis for treatment, and improve outcomes. Tsvetkov et al. found a way of cell death that differs from apoptosis, autophagy, pyroptosis, and iron death, among others; this pathway (cuproptosis) leads to cell death by copper induction [7]. The redox activity of copper participates in several enzymes’ biochemical and regulatory functions in many organisms [8]. Copper deficiency induces cell death via the associated biological functions of copper-binding enzymes and excessive accumulation of copper [9]. Mitochondrial metabolism is associated with respiratory sensitivity during cuproptosis, lipidated tricarboxylic acid (TCA) enzyme levels are increased in TCA cycle-active cells, and the fatty acyl moiety acts as a copper binder. These processes mediate aggregation of lipidated proteins, loss of Fe-S-containing cluster proteins, induction of HSP70, and, ultimately, acute proteotoxic stress [7]. FDX1 (Ferredoxin 1) and DLST (Dihydrolipoamide S-Succinyltransferase) play a crucial role in cuproptosis and affect the process of cuproptosis [7]. Non-coding RNA (ncRNA) is an area of intense interest in the medical community, and its rich biological functions stimulated enthusiasm for research and expectations for applications. ncRNAs are cancer biomarkers, and deregulated ncRNA expression has been observed in several cancers [10]. Common ncRNAs include microRNAs, long ncRNAs (lncRNAs), and circular RNAs. Of these, lncRNAs have been intensely studied in cancer research. LncRNAs include intronic lncRNAs, intergenic lncRNAs, sensory lncRNAs, antisense lncRNAs, and bidirectional lncRNAs, according to their relative positions with coding genes [11]. LncRNA-related signatures have been widely used in various tumors, such as head and neck squamous cell carcinoma [12]. LncRNAs have been widely confirmed to significantly impact biological characteristics such as the proliferation and invasion of tumors [13]. The lncRNA LINC02154 showed high expression in cells and tissues in hepatocellular carcinoma patients with poor survival; experiments demonstrated that it enhanced the invasion, migration, and proliferation of hepatocellular carcinoma cells [14]. LINC02154 has also been used to construct signatures that predict the outcomes of some tumors. A signature composed of seven immune-related lncRNAs predicted outcomes in laryngeal squamous cell carcinoma and facilitated the selection of clinical chemotherapeutic medications [15]. A novel signature consisting of four lncRNAs containing LINC02154 predicted outcomes in laryngeal cancer and regulated immunity, tumor apoptosis, metastasis, and invasion [16]. We, therefore, aimed to determine whether LINC02154 influences outcomes through pathways in ccRCC.

## Methods

### The download of clinical data and gene expression profiles

We utilized the ‘DEseq2’ R software package to obtain 1109 prognostically relevant lncRNAs, 3086 differentially expressed lncRNAs, and 484 cuproptosis-related lncRNAs in ccRCC (https://portal.gdc.cancer.gov/). We obtained gene expression profiles from 538 relevant samples from TCGA-KIRC and clinical data from 611 ccRCC patients from TCGA. We used log2 (exp + 1) and (adjusted. P-value < 0.05) to normalize differentially expressed gene data.

### Construction of ccRCC prognostic signature for cuproptosis-related lncRNAs

We used univariate Cox analysis and LASSO-penalized multivariate Cox analysis to construct ccRCC prognostic signature and then constructed the calculation formula of risk score as follows: (coefficient lncRNA1 × expression of lncRNA1) + (coefficient lncRNA2 × expression of lncRNA2) + ν + (coefficient lncRNAn × expression lncRNAn). This formula was used to calculate the risk score of each sample, which was divided into high- and low-risk groups according to the median. The individual genes of interest were divided into high-expression and low-expression groups according to the median expression level of their samples.

### Prognostic analysis of single genes and validation of risk score model

We generated Sangerbox (http://sangerbox.com/) risk charts and performed Kaplan-Meier and receiver operating characteristic analyses. Conditional survival rates were analyzed in the high-expression and low-expression groups of single genes or in high-risk and low-risk groups of risk scores to determine the validity and reliability of single genes or risk models.

### Correlation analysis of tumor mutation burden (TMB) with individual genes or risk score models

We downloaded TMB-related data from our samples from TCGA, and we analyzed the relativity between various TMB data and individual genes or risk scores using the R package *TMBcor*.

### Analysis of the immune microenvironment associated with a single gene or risk signature

We used the ESTIMATE algorithm to calculate the stromal and immune scores based on gene expression profiles. Single sample gene set enrichment analysis (ssGSEA) was used to quantify tumor-infiltrating immune cell subsets between different groups and to assess their immune functions. We also tested the relationship between single genes and our risk score and immune checkpoints. We used the *pheatmap* and *riskImmCor* packages in R to identify single genes and the correlation of risk scores with critical steps of the immune cell, immune process, and cancer immune cycle.

### Sensitivity analysis of therapeutic means

The R package *pRRophetic* was used to identify commonly used medications for ccRCC according to the gene expression matrix. The package *ggpubr* was used to draw boxplots for sensitive targets to compare the differences in sensitivity in various expression groups of LINC02154 and the high-risk and low-risk signature groups.

### Cell culture and transfection

ACHN human renal cell adenocarcinoma cells were provided by the Cell Bank of the Chinese Academy of Sciences (China). ACHN cells were cultured in MEM (Procell) containing 10% fetal bovine serum (FBS) (Gibco) at 37 ℃ and 5% CO_2_. Small interfering RNA (siRNA) for reducing LINC02154 expression was obtained from JTSBIO Co (China). The sequence of Si-LINC02154 is as follows: sense:ACCACAUUCUUUGUUGCCUGCAGUA; antisense: UACUGCAGGCAACAAAGAAUGUGGU. Cells were transfected with siRNA using LipofectamineTM3000 (Invitrogen, USA) according to the manufacturer’s guidelines.

### Quantitative real-time PCR (qRT-PCR)

We used RNAiso Plus (Takara Biotechnology, Dalian, China) to extract total RNA from cells and then reverse transcribed to synthesize cDNA using Prime Script RT Master Mix (Takara Biotechnology, Dalian, China) according to the instructions of the manufacturer. qRT-PCR was performed using the Sybr Premix Ex Taq TMKit (Takara Biotechnology, Dalian, China) and LightCyclerTM 480 II system (Roche, Basel, Switzerland). Primer sequences were as follows: Forward primer: ACTGCGCCACCTCTGATATG; Reverse primer: GACCCACTGATTGTGCCTGA.

### Cell proliferation assay

We plated ACHN cells in 96-well plates, and 2000 cells were added to each well after counting. The Cell Counting Kit-8 (CCK-8) Assay Reagent (Bimake, USA) was added to each well, and the assay was performed according to the manufacturer’s instructions. Absorbance values at 450 nm were measured on an automated microplate reader (Bio-Rad).

### EdU assay

We plated ACHN cells in 24-well plates. According to the manufacturer’s instructions, cells were treated with reagents from the EdU assay kit (Beyotime Biotechnology, China). We used a fluorescence microscope (Olympus Corporation, Japan) to obtain images, and the number of different fluorescent cells was counted using ImageJ software. The proportion of proliferating cells was then calculated.

### Cell migration assay

We used 8-µm pore Transwell chambers in 24-well plates (Corning Costar, Corning, NY, USA); 600 µL of medium containing 10% FBS were placed into each well of a 24-well plate, and 200 µL of FBS-free medium containing 10,000 suspended cells were added to each chamber. After 48 h of incubation at 37 °C in 5% CO_2_, cells suspended in the chambers were washed out with phosphate-buffered saline, and cells adhering to the bottom membrane were stained using crystal violet. Images were obtained at 10X magnification using an inverted microscope (EVOS XL system, AMEX1200; Life Technologies Corp, Bothell, WA, USA), and cell counts were performed using Image J.

### Wound-healing assay

When the density of ACHN cells reached more than 90% in six-well plates, we scratched an artificial wound in the middle of the well using a 200-µL pipette tip. FBS-free medium was added to each well after washing with phosphate-buffered saline. Images were taken at 10x using the inverted microscope as previously described. After incubation for 24 h, images were retaken.

### Western blot

After washing the cells with PBS, we treated the cells with RIPA lysate containing 1% PMSF, extracted the protein, and then determined the protein concentration using the bicinchoninic acid assay kit. We used PAGE and polyvinylidene fluoride membranes for electrophoresis and transfer. We used FDX1 (1:1000, 12592-1-AP, proteintech, China), DLST (1:1000, 11,954, CST, USA), GAPDH (1:1000, 5174, CST, USA), antibodies for incubation at 4 ° C. Following overnight washing with TBST three times, membranes were incubated with secondary antibodies for one hour at 37 ° C. The EasySee Western Blot kit (Beijing Genetically Modified Biotechnology Co., Ltd., Beijing, China) was then used for imaging, and the chemiluminescence system (Bio-Rad), California, USA) was used to acquire images.

## Results

### Construction of ccRCC prognostic risk signature by cuproptosis-related genes and differentially expressed genes

**Fig. 1:**
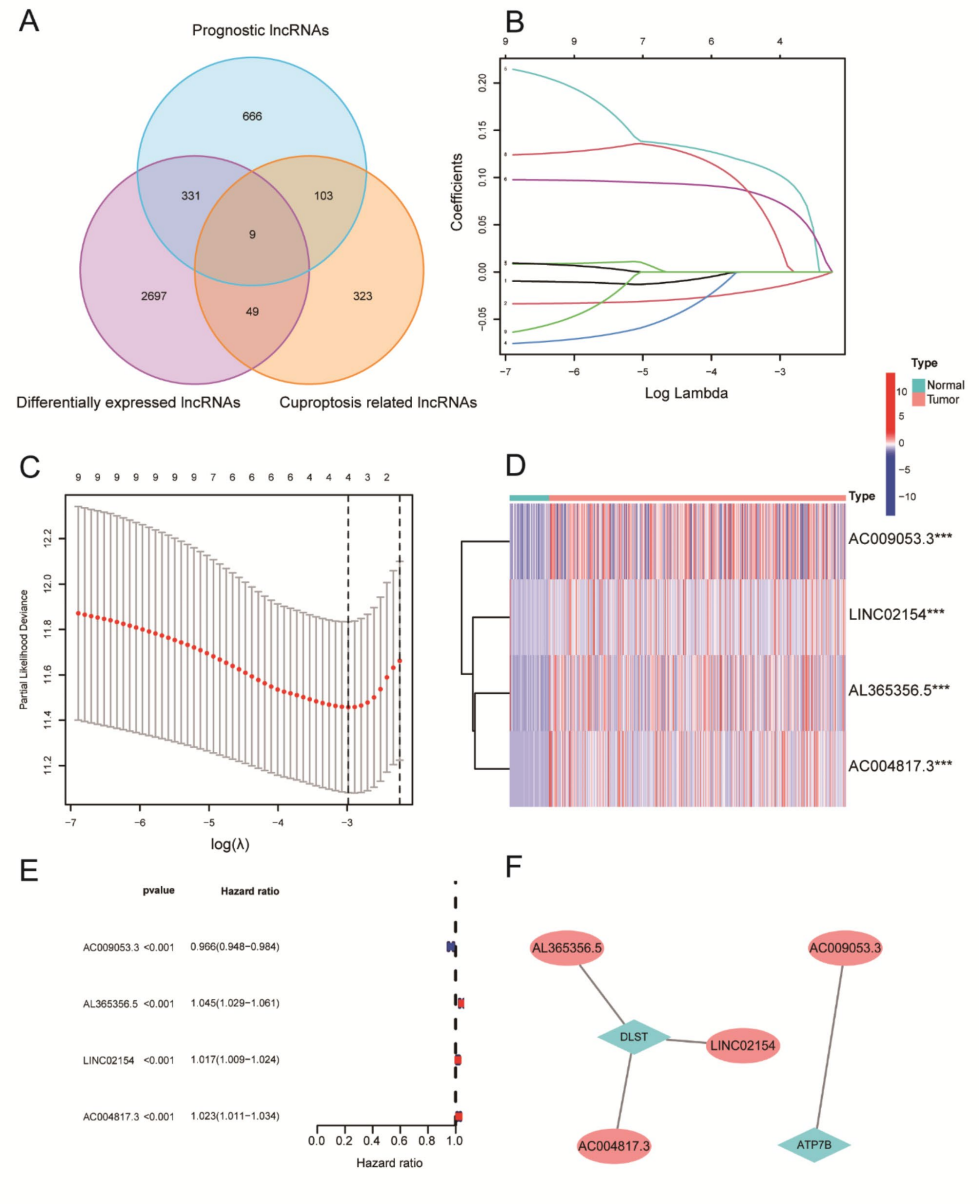
Selection of cuproptosis-related lncRNAs. **A** Relationship and number of prognostic lncRNAs differentially expressed lncRNAs and cuproptosis-related lncRNAs. **B** Least absolute shrinkage and selection operator coefficient spectra of nine ccRCC prognostic genes. **C** Optimal lambda. **D, E** Differential expression levels of these four genes in ccRCC versus normal tissues. **F** Correlation of four selected genes with cuproptosis key genes

We obtained 1109 lncRNAs with close relativity in the outcomes of ccRCC, 3086 differentially expressed lncRNAs, and 484 cuproptosis-related lncRNAs from TCGA using the R package. We obtained nine genes by intersecting the three lncRNAs (Fig. 1A). We used univariate Cox analysis to identify nine genes significantly associated with prognostic levels in ccRCC and then performed LASSO-penalized multivariate Cox analysis of these nine genes. Finally, we established a risk prediction signature consisting of four genes (Fig. 1B and C). The specific calculation formula was as follows:

Risk score =-0.0147356703938245 * AC009053.3 + 0.101615765203553 * AL365356.5 + 0.0724570066296659 * LINC02154 + 0.0254437556729956 * AC004817.3.

Using statistical analysis, we discovered that these four genes significantly differed in expression levels between ccRCC and normal tissues (Fig. 1D and E). We correlated the four selected genes with cuproptosis genes and found that the risk signature was correlated with DLST and ATP7B (Fig. 1F). Using TCGA, we constructed training and testing sets to validate the reliability of our risk signature.

**Fig. 2:**
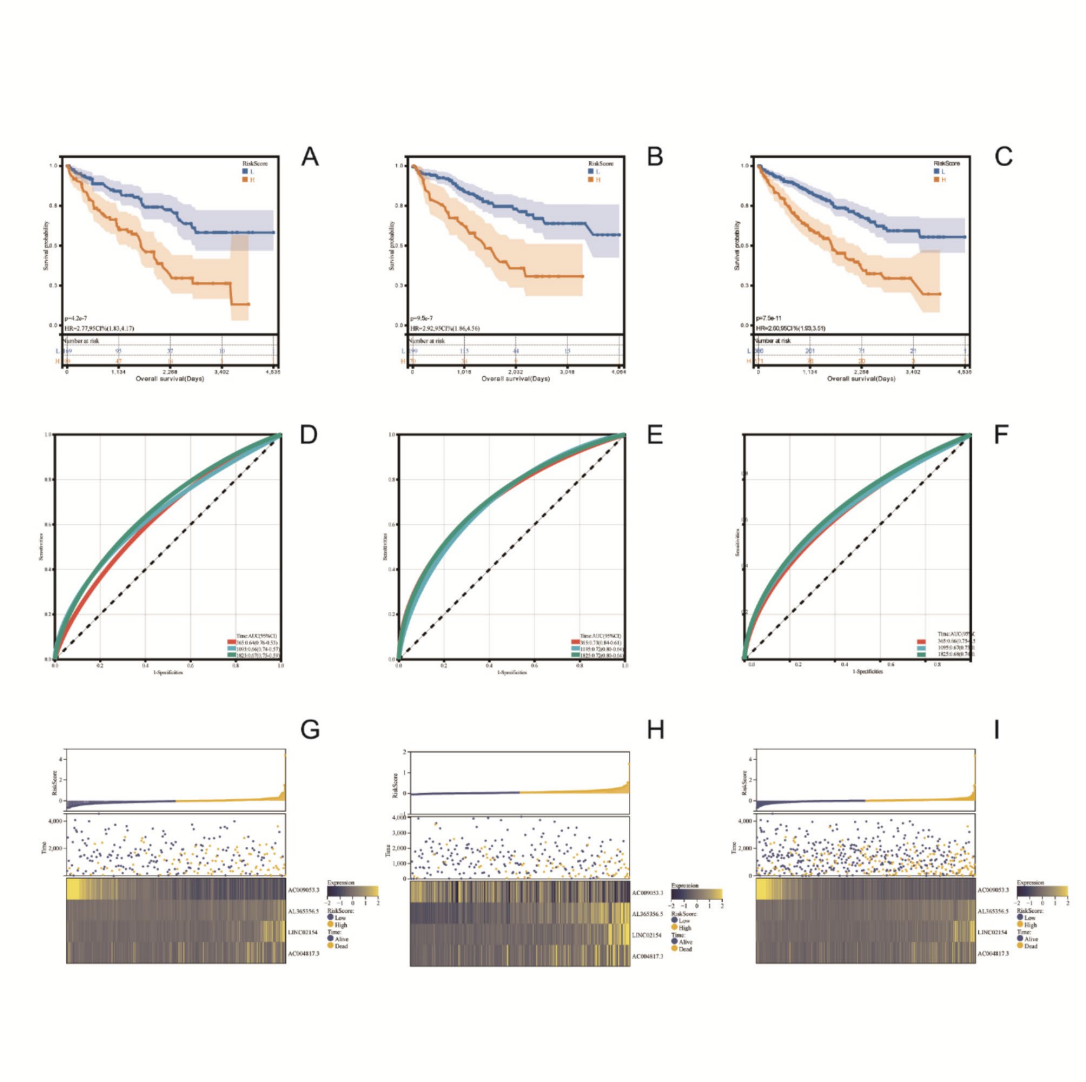
Prognostic differences by risk scores. **A-C** Kaplan-Meier analysis showed significant differences in five-year overall survival between high-risk and low-risk groups in the training, testing, and full sets. **D-F** Receiver operating characteristic curves for overall survival at 1, 3, and 5 years in the training, testing, and full sets. **G-I** Risk scores were ranked from low to high to obtain patient and expression heatmaps for four genes in The Cancer Genome Atlas training, testing, and full sets

We found a statistically significant difference in five-year overall survival (OS) between the high- and low-risk groups distinguished by the risk signature in the training, testing, and full sets (Fig. 2A-C). We measured the area under the curve (AUC) to validate the impact of the risk signature on survival. In the training set, the area under the ROC curve was 0.64 for one-year OS, 0.66 for three-year OS, and 0.67 for five-year OS (Fig. 2D). In the testing set, the AUC was 0.73 for one-year OS, 0.72 for three-year OS, and 0.72 for five-year OS (Fig. 2E). In the full set, the AUC was 0.66 for one-year OS, 0.67 for three-year OS, and 0.68 for five-year OS (Fig. 2F). Heatmap analysis showed that there were significant differences in the expression levels of these four genes used to construct the risk signature in the high-risk and low-risk groups, whether in the training, testing, or full sets (Fig. 2G-I).

**Fig. 3:**
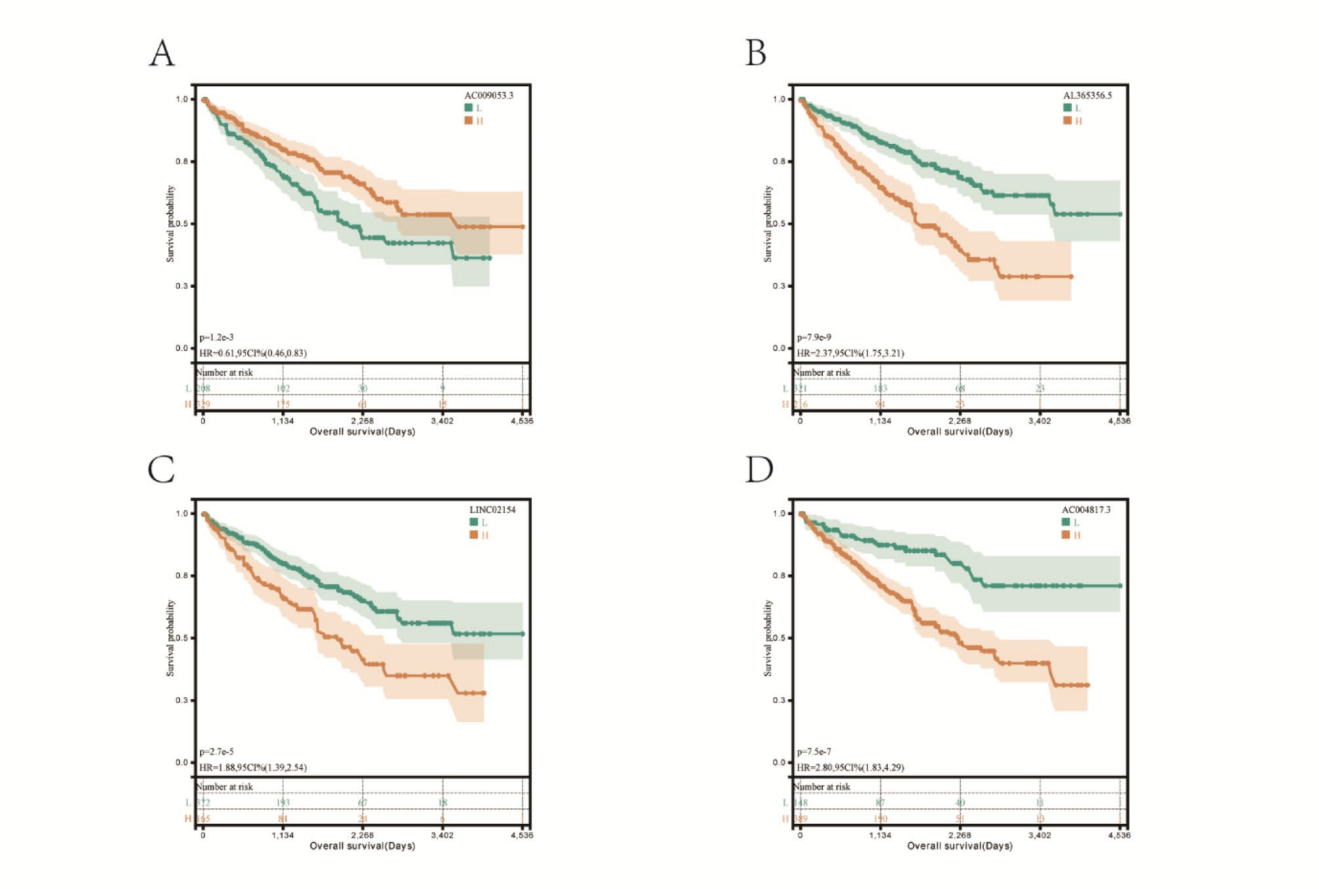
A single-gene survival analysis was performed on all four genes in the risk signature in the full set

We performed single-gene survival analysis on the four genes in the risk signature in the full set and found that the expression differences independently affected survival (Fig. 3A-D).

We performed the same single-gene survival analysis on the four genes in the training and testing sets and obtained the same results as the full set. All four genes independently and significantly affected survival (Supplementary Figures S1 and 2). Univariate and multivariate Cox regression analyses showed that age, grade, stage, and risk score independently predicted outcomes in ccRCC (Table 1). Next, we wanted to build a nomogram combining clinical parameters and risk scores, thereby improving the predictive efficiency of the model. The predictive efficiency of the nomogram can be improved by excluding non-significant clinical parameters (Gender).

**Table 1:** Univariate and multivariate Cox regression analyses of age, gender, grade, age, and risk score

Variables	Univariable analysis				Multivariable analysis			
	HR	95% CI of HR		P	HR	95% CI of HR		P
		lower	upper			lower	upper	
Age	1.03	1.01	1.04	2.18E-05	1.03	1.01	1.04	6.08E-05
Gender	0.97	0.71	1.33	0.8675	0.89	0.64	1.24	0.6315
Grade	2.26	1.84	2.77	4.43E-15	1.11	0.27	4.63	0.0040
Stage	1.86	1.63	2.13	2.46E-20	1.66	1.36	2.02	4.33E-11
RiskScore	4.52	3.08	6.62	1.07E-14	7.71	3.04	19.56	2.74E-07

We built a nomogram based on the information of clinical parameters (Age, Grade, Stage) and risk score for prediction of prognosis (Supplementary Figure S8A), and calibration was for identification of accuracy and reliability (Supplementary Figure S8B). The AUCs demonstrated a better efficiency of the nomogram than the risk model and each clinical parameter (Supplementary Figure S8C).

**Fig. 4:**
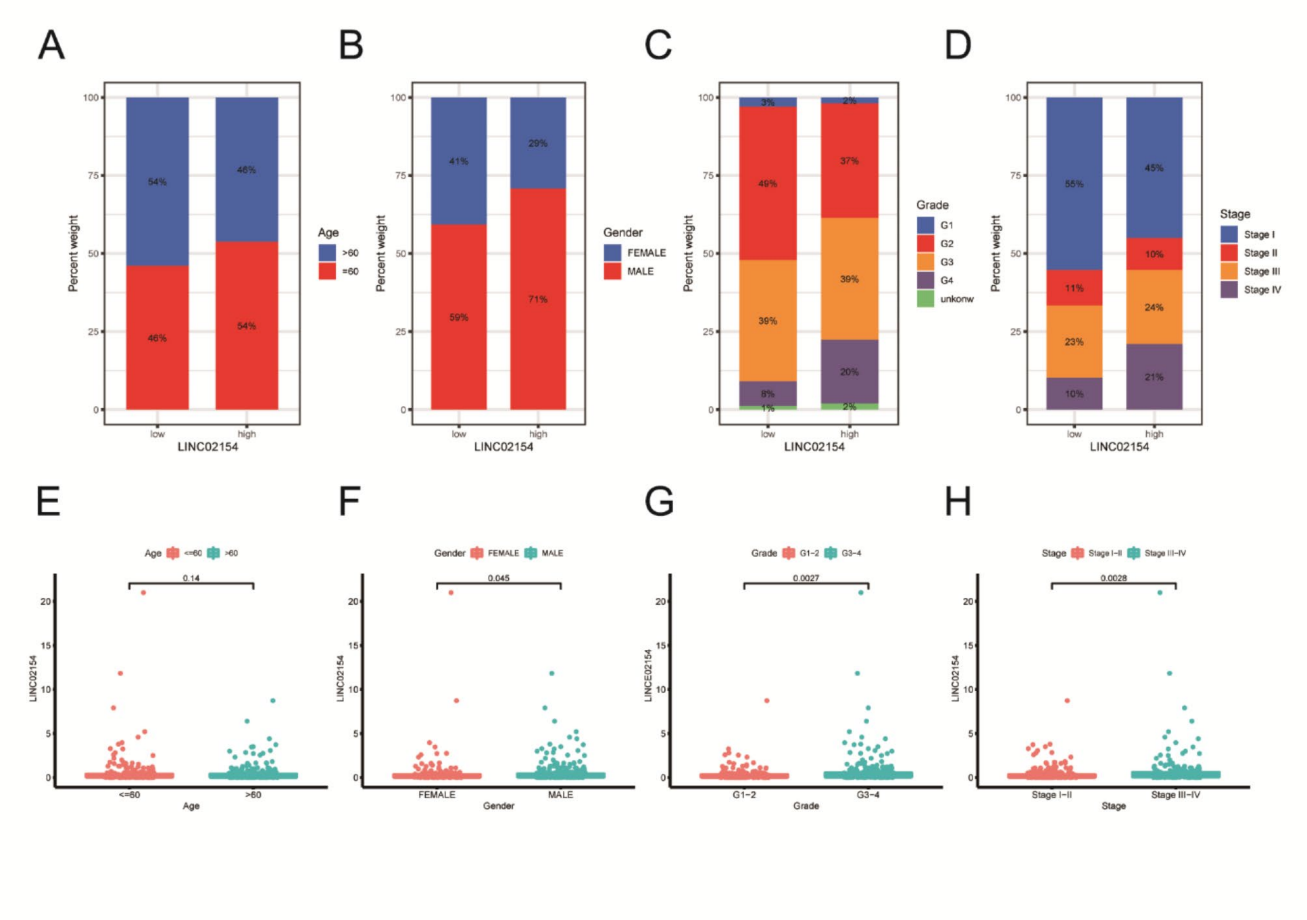
The relationship between age, gender, stage, grade, and LINC02154. **A-D** Different proportions of age, gender, stage, and grade were observed between the high LINC02154 expression group and the low LINC02154 expression group. **E-H** Expression levels of LINC02154 across ages, genders, stages, and grades

We analyzed the proportions of age, gender, stage, and grade in the high- and low-expression groups of LINC02154 (Fig. 4A-D) and found that expression levels of LINC02154 varied across ages, genders, stages, and grades (Fig. 4E-H).

**Fig. 5:**
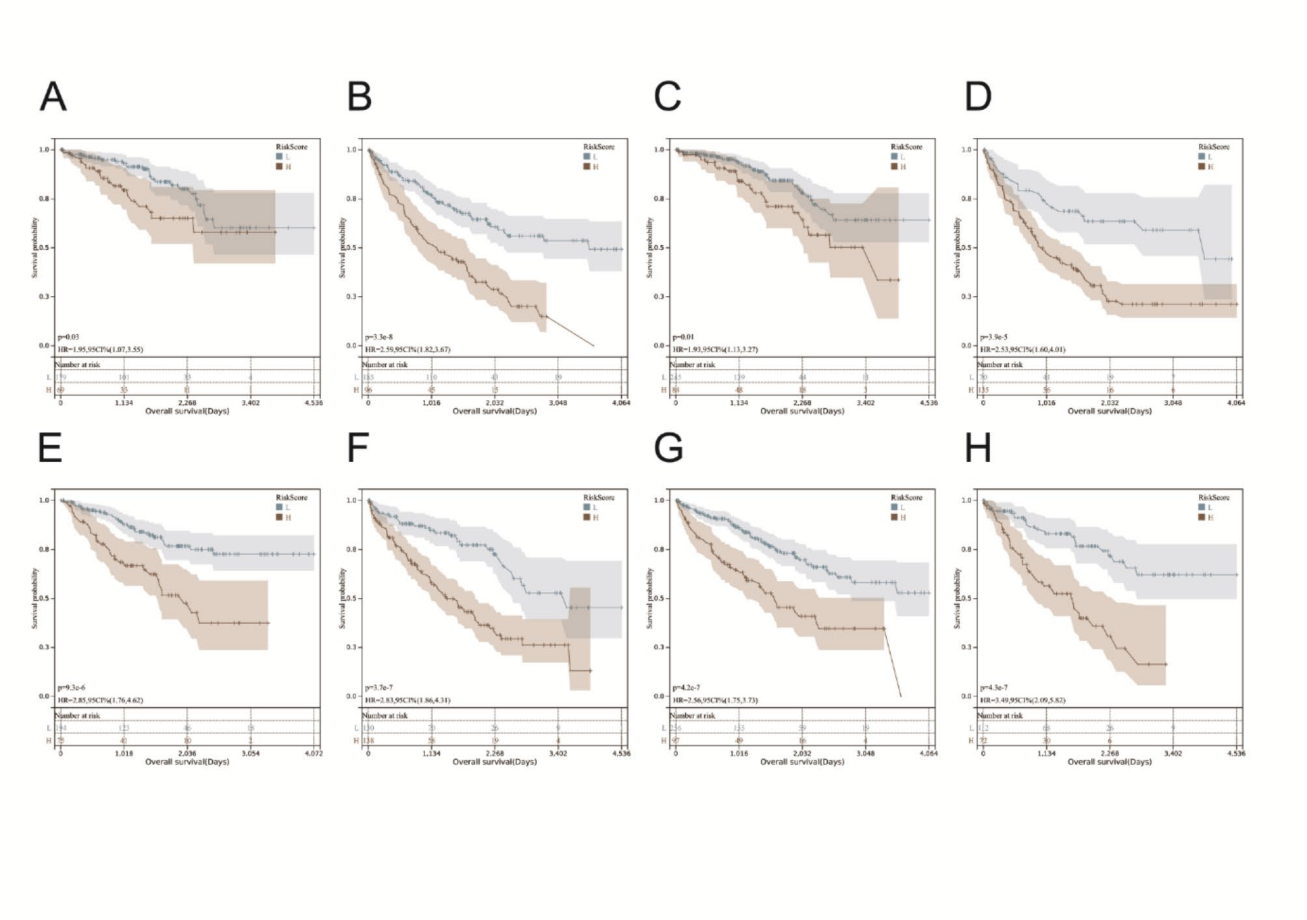
The relationship between age, gender, stage, grade, and patient prognosis. **A-B** In the full set, the grade was significantly associated with outcomes. **C-D** In the full set, the stage was associated with the outcome significantly. **E-F** In the full set, age was associated with outcome significantly. **G-H** In the full set, gender was associated with outcome significantly

We then found significant correlations between our constructed risk signature and age, gender, stage, and grade (Supplementary Figure S3). We divided patients into groups according to their grade (Grade 1 + Grade 2 and Grade 3 + Grade 4) and assessed risk signature scores in each group. The risk scores were significantly associated with patient outcomes in both groups (Fig. 5A and B). We then divided patients into groups according to the stage (Stage 1 + Stage 2 and Stage 3 + Stage 4) and evaluated the risk signature score in each group. Our risk model significantly correlated with outcomes (Fig. 5C and D). We then divided patients into groups according to age (60 years or older and less than or equal to 60 years) and assessed the risk signature score in each group. The risk model significantly correlated with the outcomes (Fig. 5E and F). We then grouped patients according to gender and assessed outcomes in high-risk and low-risk groups. Our risk model significantly correlated with outcomes (Fig. 5G and H).

We validated the correlation analysis of these subclinical groupings in the training and testing sets and achieved similar results as in the full set (Supplementary Figures S4 and 5).

### Correlative analysis of tumor mutation burden

**Fig. 6:**
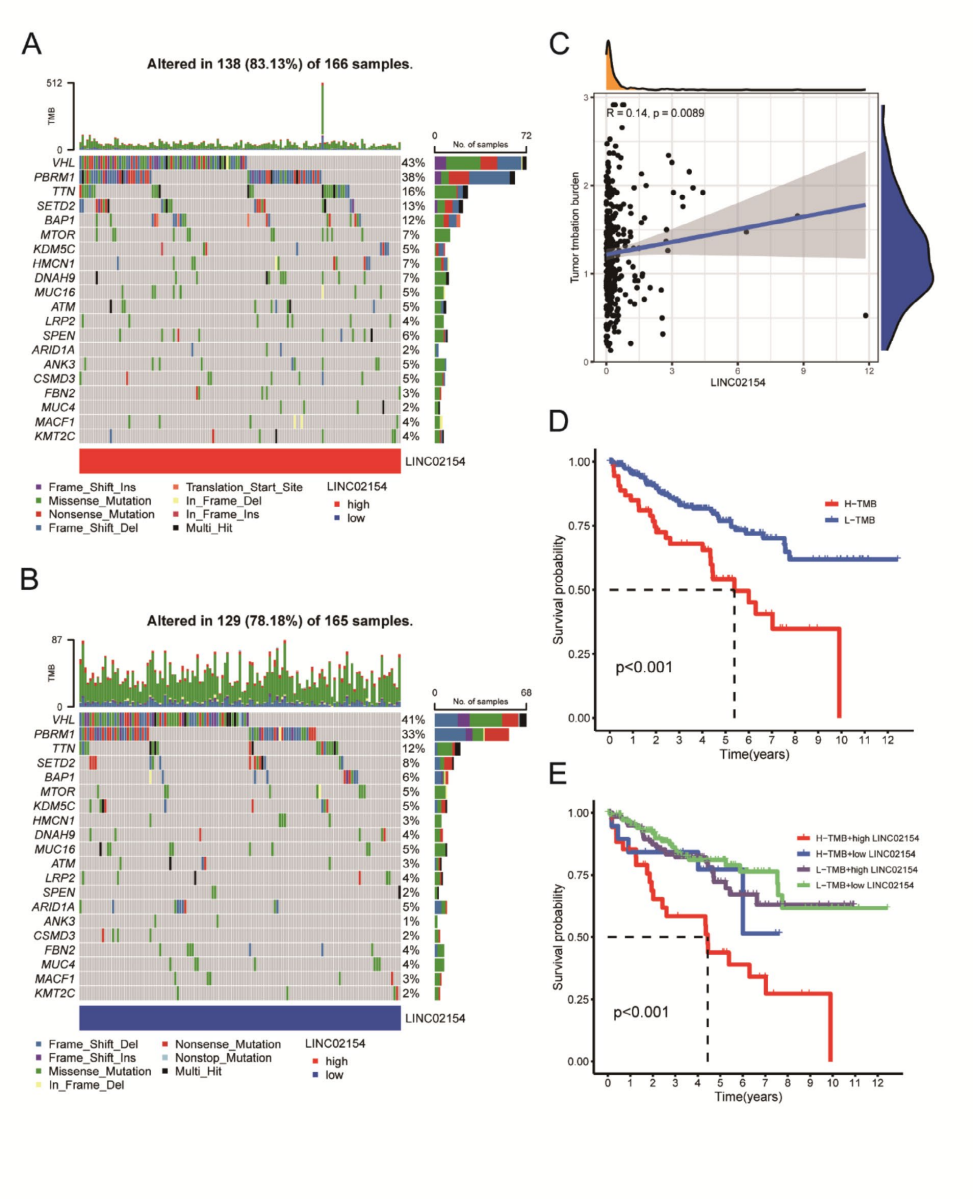
The relationship between different LINC20154 expression levels, tumor mutation burden, and patient survival outcomes. **A** Tumor-associated gene mutation burden in samples from the high LINC02154 expression group. **B** Tumor-associated gene mutation burden in samples from the low LINC02154 expression group. **C** Positive relationship between LINC02154 expression and tumor mutation burden. **D** Survival analysis of high and low tumor mutation burden groups. **E** Survival analysis of H-TMB + high LINC02154 group, H-TMB + low LINC02154 group, L-TMB + high LINC02154 group, and L-TMB + low LINC02154 group

We analyzed TMB in the high LINC02154 expression group and low LINC02154 expression group and found a significant difference in the TMB ((Fig. 6A and B). We also found a positive relationship between LINC02154 expression and TMB (Fig. 6C). We performed survival analysis in the high and low tumor mutation load groups. We found that the high TMB group had significantly worse outcomes than the low TMB (Fig. 6D). We then created four groups according to TMB: H-TMB + high LINC02154, H-TMB + low LINC02154, L-TMB + high LINC02154, and L-TMB + low LINC02154. We performed survival analysis and found that high TMB and high LINC02154 predicted poor survival (Fig. 6E).

We analyzed the correlation between our risk signature and TMB and found significant differences between the high-risk and low-risk groups and TMB (Supplementary Figure S6A and B). Our risk signature also positively correlated with TMB (Supplementary Figure S6C) survival analysis showed that high tumor mutation load preconditioned poor survival outcomes (Supplementary Figure S6D). We then created four groups according to the level of tumor mutation load and the level of risk: H − TMB + high-risk, H − TMB + low-risk, L − TMB + high-risk, and L − TMB + low-risk. We performed survival analysis and found that high tumor mutation load or high risk predicted poor survival outcomes (Supplementary Figure S6E).

### Analysis of the immune microenvironment

**Fig. 7:**
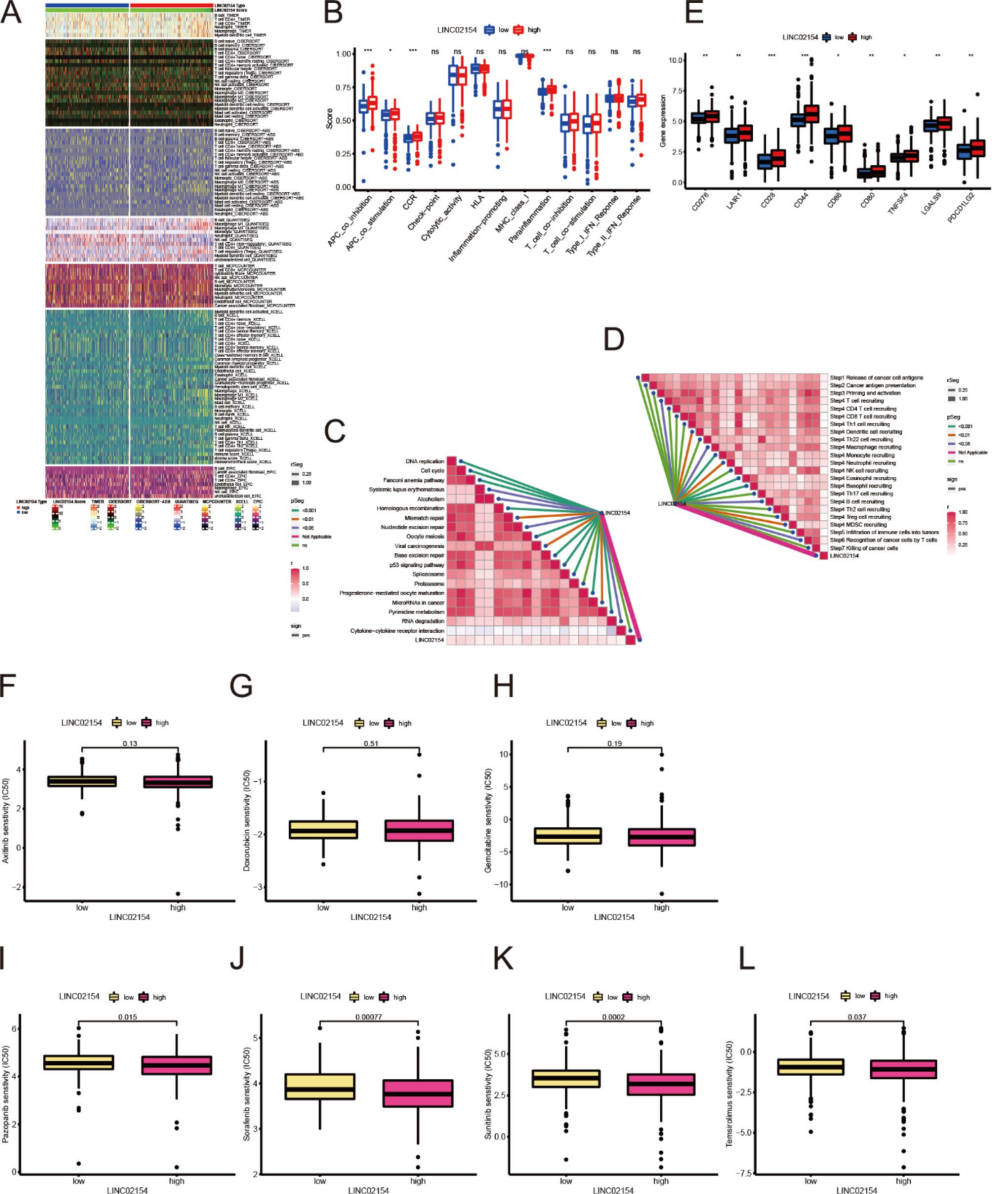
LINC02154 immune microenvironment associated analysis and drug sensitivity analysis. **A** Relationship between LINC02154 expression type and immune molecular typing. **B** Analysis of immune function in high LINC02154 and low LINC02154 expression groups. **C** Correlation analysis between LINC02154 and positive markers related to immunotherapy. **D** Analysis of the correlation between LINC02154 and critical steps of the cancer immunity cycle. **E** Immune checkpoint analysis of high LINC02154 and low LINC02154 expression groups. **F-L** Susceptibility analysis was performed separately for axitinib, doxorubicin, gemcitabine, pazopanib, sorafenib, sunitinib, and temsirolimus in high LINC02154 expression group and low LINC02154 expression group

We performed a correlation analysis of the immune microenvironment in the high LINC02154 expression group and low LINC02154 expression group and obtained a heat map of expression (Fig. 7A). LINC02154 expression significantly correlated with immune cell expression. We scored and compared immune function according to the expression of LINC02154 and found that the immune function of the high LINC02154 expression group was more active than that of the low LINC02154 expression group (Fig. 7B). We performed enrichment scoring against key steps of immunotherapy and found that LINC02154 had a positive relationship with the positive markers in immunotherapy (Fig. 7C). We also found a positive correlation between critical steps in the tumor immune cycle and LINC02154 (Fig. 7D). We performed a correlation analysis of immune checkpoints. We found that CD276, CAIR1, CD28, CD44, CD86, CD80, TNFSF4, LGALS9, and PDCD1LG2 were significantly higher in the high LINC02154 expression group than in the LINC02154 expression group (Fig. 7E).

We performed correlation analysis for the immune microenvironment and found significant differences between the high-risk group and low-risk group of the risk signature using an expression heat map (Supplementary Figure S7A). We compared immune function in the high-risk and low-risk groups according to the risk signature and found that immune function was more active in the high-risk group than in the low-risk group (Supplementary Figure S7B). We performed enrichment scoring of critical immunotherapy steps and found that our risk signature positively correlated with positive markers associated with immunotherapy and critical steps of the tumor immune cycle (Supplementary Figure S7C and D). We performed a correlation analysis of immune checkpoints and found that PDCD1, TMIGD2, TNFSF14, TNFRSF18, CD44, LGALS9, and LAG3 were significantly higher in the high-risk group than in the low-risk group (Supplementary Figure S7E).

### Medication sensitivity analysis

We performed a medication sensitivity analysis of axitinib, doxorubicin, gemcitabine, pazopanib, sorafenib, sunitinib, and temsirolimus, which are commonly used medications for ccRCC. We found that the sensitivities of pazopanib, sorafenib, sunitinib, and temsirolimus in the low-expression group of LINC02154 were significantly higher than those in the high-expression group (Fig. 7F-L). This finding suggests that the expression level of LINC02154 might guide clinical use. We performed a sensitivity analysis of doxorubicin, gemcitabine, pazopanib, sorafenib, sunitinib, and temsirolimus in the high- and low-risk groups and found that there were statistically significant differences in medication sensitivity, especially the sensitivities of doxorubicin, gemcitabine, and sorafenib, which were significantly higher in the high-risk group than in the low-risk group. For sunitinib, sensitivity was significantly higher in the low-risk group than in the high-risk group (Supplementary Figure S7F-K).

### Knockdown of LINC02154 significantly inhibited the proliferation and migration of ccRCC cells and affected cuproptosis

**Fig. 8:**
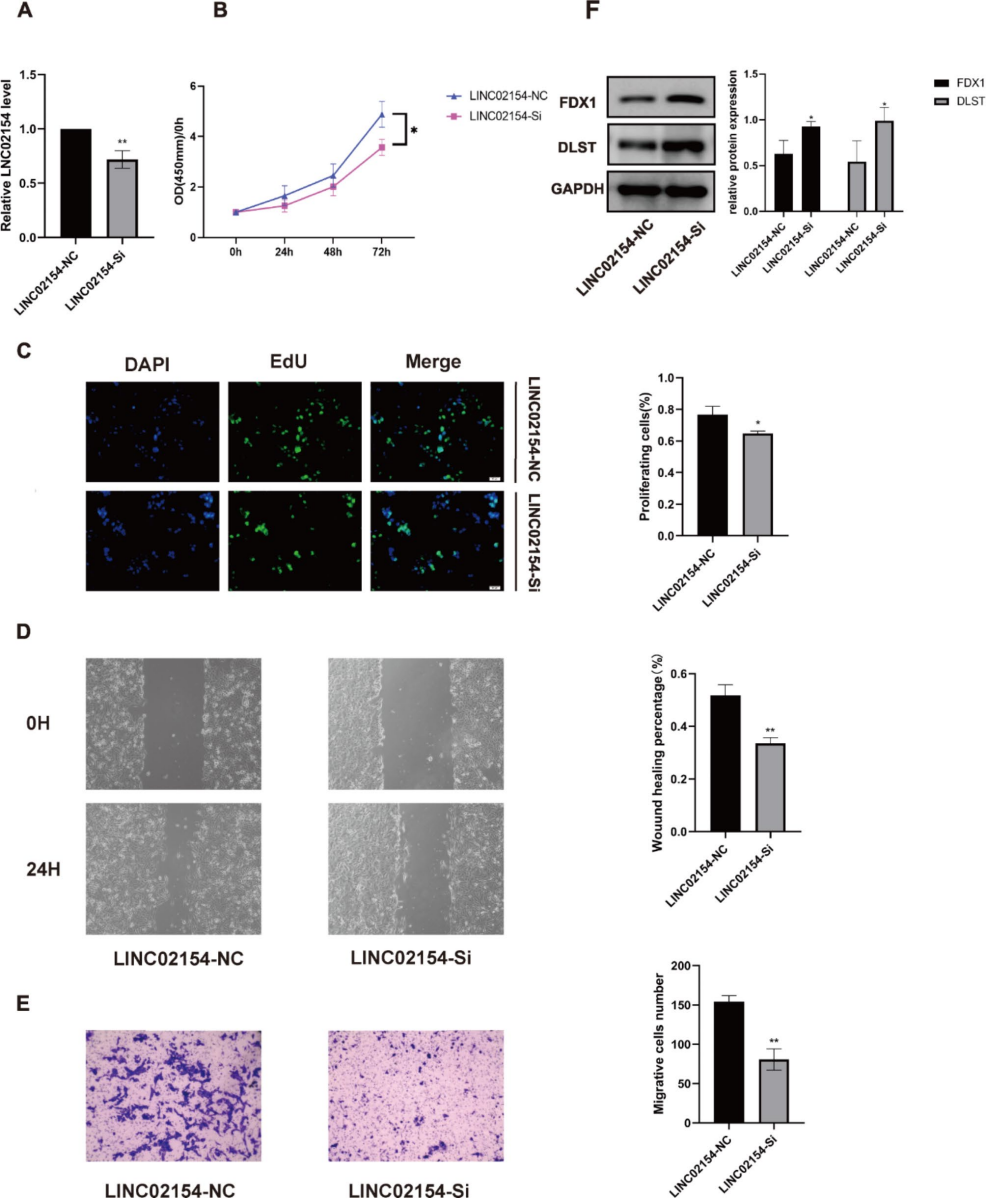
Functional experiments against LINC02154 in ACHN cells. **A** Relative expression levels of LINC02154 after transfection with the corresponding siRNA. **B** CCK-8 assay was used to detect the effect of LINC02154 on ACHN cell proliferation. **C** The effect of LINC02154 on ACHN cell proliferation was examined by EdU assay. **D** Wound-healing assay was used to test the effect of LINC02154 on ACHN cell migration. **E** The Transwell assay was used to test the effect of LINC02154 on ACHN cell migration. F Western blot indicated that FDX1 and DLST were significantly upregulated in the LINC02154-Si group relative to the LINC02154-NC group

We performed functional experiments against LINC02154 in ACHN cells. First, we designed siRNA strands targeting LINC02154 and measured knockdown levels using qRT-PCR (Fig. 8A). We then performed a CCK-8 assay to measure viability and found ACHN viability was significantly decreased after transfection with siRNA (Fig. 8B). We also performed EdU assay. We found that proliferation after the knockdown of LINC02154 was significantly decreased (Fig. 8C). Knockdown of LINC02154 resulted in a statistically significant decrease in the wound-healing rate of ACHN cells (Fig. 8D). Transwell assays showed a statistically significant decrease in the cell migration after LINC02154 knockdown (Fig. 8E). Western blot experiments showed that knockdown of LINC02154 upregulated the expression of cuproptosis critical genes FDX1 and DLST(Fig. 8F).

## Discussion

Changes in tumor energetics and biosynthetic metabolic pathways are essential for tumor research. In ccRCC, there is a reprogramming of glucose and fatty acid metabolism and changes in the metabolism of the tricarboxylic acid cycle, tryptophan, arginine, and glutamine; for these reasons, the exploration of the mechanisms and factors influencing metabolism in ccRCC are promising [17]. LncRNAs regulate processes such as energy metabolism in cancer, and a deep understanding of lncRNA-mediated cancer metabolic reprogramming can be beneficial for the diagnosis and treatment of cancer [18]. Copper increases mitochondrial protein fatty acylation, regulates carbon entry into the TCA cycle, and binds dihydrolipoamide S-acetyltransferase (DLAT) to promote disulfide-dependent aggregation of fatty acylated DLAT [19]. Ferredoxin 1 (FDX1) is a lipid-acylation effector, contributing to the accumulation of toxic lipid-acylated DLAT and cuproptosis. FDX1-dependent degradation of Fe-S cluster proteins might facilitate cuproptosis [19]. We, therefore, analyzed lncRNAs associated with ccRCC outcomes, differentially expressed lncRNAs, and cuproptosis-associated lncRNAs and established a risk prediction signature constituted by four genes. According to the risk signature, survival analysis of the high and low groups revealed significant differences. Survival analysis of the four single genes showed that all independently and significantly predicted survival outcomes. Based on the literature, we focused on LINC02154 for in-depth analysis. LINC02154 and the risk signature were analyzed separately in subclinical groupings of age, gender, stage, and grade, and all showed significant differences. TMB can identify patients who may respond to immune checkpoint blockade and discern the type and extent of TMB variants across tumor types and histologies [20]. High TMB is associated with the appearance of tumor neoantigens on HLA molecules on the surface of tumor cells with a high probability [21]. Therefore, we speculated that a high TMB might predict a higher effectiveness of targeted therapy. Our TMB analysis of LINC02154 showed a significant difference in TMB between the high and low LINC02154 expression groups, with high LINC02154 expression often predicting higher TMB and high TMB predicting poorer survival. In the same way, we applied the risk signature and found that a high-risk score predicted a high TMB and poor survival. The tumor immune microenvironment is essential for identifying immune modifiers of cancer progression and developing cancer immunotherapies [20]. We performed a heat map analysis of the immune microenvironment and related assessments of immune function against LINC02154 and found that high expression of LINC02154 changes the immune microenvironment and makes the immune function more active. LINC02154 can be positively correlated with positive markers in immunotherapy and key steps of the immune cycle, which predicts that LINC02154 can play a role in immunotherapy. Analysis of immune checkpoints showed that CD276, CAIR1, CD28, CD44, CD86, CD80, TNFSF4, LGALS9, and PDCD1LG2 were more highly expressed in the high LINC02154 expression group than in the low LINC02154 expression group. We also performed an associated immune microenvironment analysis for the risk signature and found a similar trend to LINC02154. Regarding immune checkpoints, PDCD1, TMIGD2, TNFSF14, TNFRSF18, CD44, LGALS9, and LAG3 expression were higher in the high-risk group than in the low-risk group.Seaman et al. demonstrated that the cell surface protein CD276/B7-H3 is overexpressed in several cancers and tumor-infiltrating vessels; CD276 antibody-medication conjugates (ADCs) equipped with conventional monomethyl auristatin E warheads could kill CD276-positive cancer cells but have little effect on tumor vasculature. Pyrrolobenzodiazepine-conjugated CD276 ADCs can kill cancer cells and tumor vasculature, eradicate large tumors and metastases, and improve OS [22]. CAIR-1/BAG-3 may serve as a multifunctional signaling protein linking the pathways necessary for activating the EGF receptor tyrosine kinase signaling pathway to the Hsp70/Hsc70 pathway [23]. CD28 signaling plays a critical role in many biological processes of T cells, including cytoskeletal remodeling, cytokine production, survival, and differentiation and CD28 not only acts as an amplifier of TCR signaling but also can be the source of unique signals and regulates intracellular biochemical processes, including post-translational protein modification and epigenetic changes [24]. CD44 is a non-kinase transmembrane glycoprotein, and its primary ligand is hyaluronic acid, which can be activated after binding to it, and then activate cell signaling pathways, promote cell proliferation, regulate the cytoskeleton, and enhance cell viability. CD44 is overexpressed in cancer stem cells, and a study suggested that alternatively spliced variants participate in tumor progression [25]. CTLA-4 is a negative modulator of T cell immune responses, which shares two ligands (CD80 and CD86) with the stimulatory receptor CD28. CTLA-4 captures its ligand from opposing cells by trans-endocytosis [26]. TNFSF4 was significantly upregulated in lung fibroblasts exposed to stress, and there was a negative correlation between TNFSF4 and tumor shrinkage after treatment with chemotherapeutic agents [27]. Glioblastoma multiforme-derived exosome LGALS9 can play a significant regulatory role in tumor progression by inhibiting dendritic cell antigen presentation and cytotoxic T cell activation in CSF; if this inhibition is lost, it can lead to long-lasting systemic anti-tumor immunity [28]. Masugi et al. found a negative correlation between PDCD1LG2 expression and Crohn’s-like lymphoid response in colorectal cancer, suggesting that PDCD1LG2 positive tumor cells may be involved in inhibiting the development of tertiary lymphoid tissues during colorectal carcinogenesis [29].We performed susceptibility-related analysis and found that differential expression of LINC02154 affected the sensitivity of pazopanib, sorafenib, sunitinib, and temsirolimus. Moreover, there were significant differences in sensitivity to doxorubicin, gemcitabine, sorafenib, and sunitinib between the high- and low-risk score groups. Pazopanib is an oral angiogenesis inhibitor. A randomized, double-blind, placebo-controlled phase III study investigated the safety and efficacy of pazopanib monotherapy in cytokine-pretreated advanced renal cell carcinoma; the authors showed the efficacy of pazopanib in patients with advanced and metastatic RCC reflected improved tumor response and progression-free survival compared with placebo [30]. Sorafenib was the first multikinase inhibitor approved to treat RCC in the US and Europe [31]. Several studies demonstrated the efficacy of sunitinib in patients with metastatic RCC [32–35]. A phase III clinical trial showed that temsirolimus had a significantly better treatment effect than IFN-α treatment in RCC patients with poor outcomes in terms of OS, progression-free survival, and tumor response [36].

In summary, we analyzed the role of LINC02154, a gene associated with cuproptosis-related lncRNAs, in ccRCC. A risk signature composed of four genes for ccRCC containing LINC02154 was constructed, which has clinical value for the outcomes of ccRCC and the judgment of the benefit of targeted therapy and neoadjuvant chemotherapy. We also investigated the proliferation and migration of LINC02154 using cell, CCK-8, EdU, wound-healing, and Transwell assays. We found that the knockdown of LINC02154 inhibited the proliferation and migration of ccRCC cells. Many LINC02154-related data were analyzed using bioinformatics and experimentally verification; nevertheless, there were limitations, Except for TCGA, we did not find the expression information of LINC02154 in the RNA matrix of other patient cohorts, so we could not verify it in other patient cohorts. Besides, more in-depth animal experiments and clinical experiments must be performed to promote the application of LINC02154 in clinical work.

## Conclusion

We analyzed the related genes of copper death, constructed a cuproptosis-related prognostic risk signature from four lncRNAs, and validated the risk signature. LINC02154 was differentially expressed across ages, genders, stages, and grades. Various expression levels did affect outcomes and had a significant relationship with TMB and medication sensitivity. We performed cell function experiments and showed that LINC02154 knockdown significantly predicted the proliferation and migration of ACHN cells. These findings suggest that LINC02154 can predict ccRCC outcomes and has potential clinical application value.

## Electronic supplementary material

Below is the link to the electronic supplementary material.

Supplementary Material 1

Supplementary Material 2

Supplementary Material 3

Supplementary Material 4

Supplementary Material 5

Supplementary Material 6

Supplementary Material 7

## Data Availability

The TCGA-KIRC set used in this study could be obtained from the TCGA database (https://cancergenome.nih.gov/).

## Abbreviations

ccRCC: Clear cell renal cell carcinoma
LncRNA: Long non-coding RNA
TCGA: The Cancer Genome Atlas
TCA: Tricarboxylic acid
NcRNA: Non-coding RNA
ROC: Receiver operating characteristic
TMB: Tumor mutation burden
ssGSEA: single sample gene set enrichment analysis
qRT-PCR: Quantitative real-time PCR
DLST: Dihydrolipoamide S-Succinyltransferase
ATP7B: ATPase Copper Transporting Beta
OS: Overall survival
AUC: Area under the curve
TNFSF4: Tumor Necrosis Factor (Ligand) Superfamily, Member 4
LGALS9: Lectin, Galactoside-Binding, Soluble, 9
PDCD1LG2: Programmed Cell Death 1 Ligand 2
PDCD1: Programmed Cell Death 1
TMIGD2: Transmembrane And Immunoglobulin Domain Containing 2
TNFSF14: Tumor Necrosis Factor (Ligand) Superfamily, Member 14
TNFRSF18: TNF Receptor Superfamily Member 18
LAG3: Lymphocyte-Activation Gene 3
DLAT: Dihydrolipoamide S-acetyltransferase
FDX1: Ferredoxin 1
ADCs: Antibody-medication conjugates
EGF: Epidermal Growth Factor
Hsp70/Hsc70: Heat Shock Protein Family A
TCR: T cell receptor
CTLA-4: Cytotoxic T-Lymphocyte Associated Protein 4
PDCD1LG2: Programmed Cell Death 1 Ligand 2
IFN-α: Interferon Alpha
