# Supplementary material for: Bioinformatics analysis and experimental validation of cuproptosis-related lncRNA LINC02154 in clear cell renal cell carcinoma
Source: BMC Cancer. 2023 Feb 16;23:160. doi: 10.1186/s12885-023-10639-2 (PMC9936708; doi:10.1186/s12885-023-10639-2)
Supplement: Supplementary file 1 — Supplementary Material 1 [file 12885_2023_10639_MOESM1_ESM.pdf]

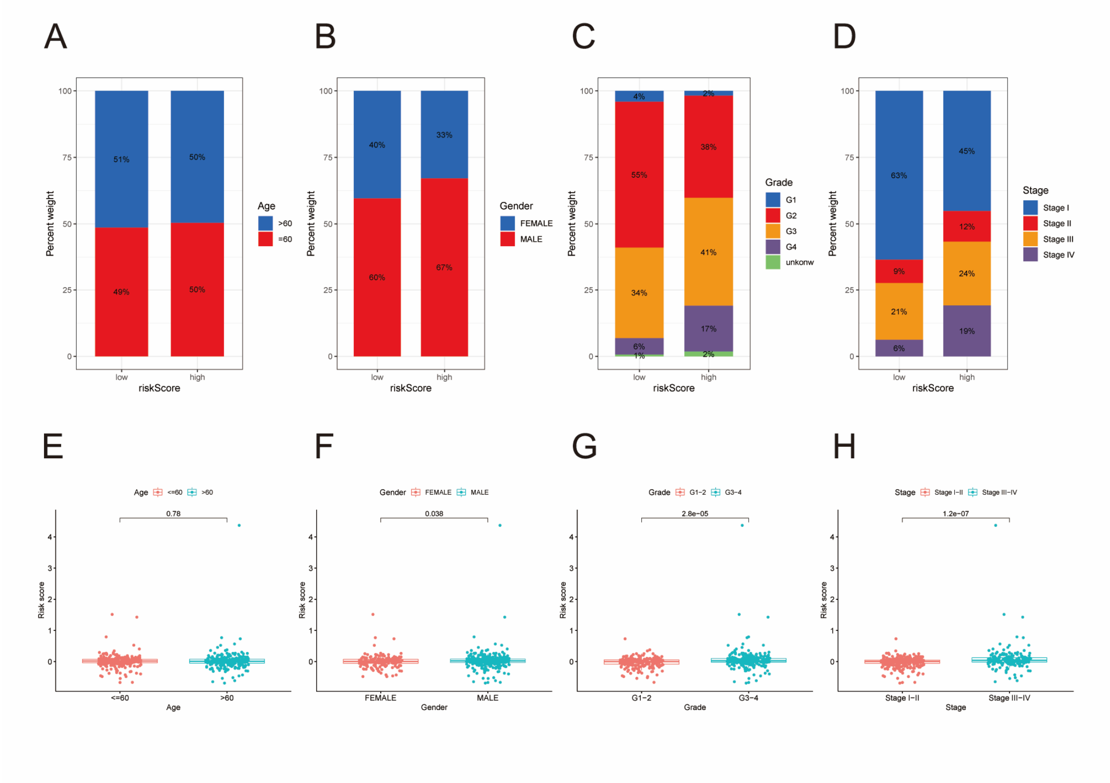

Supplementary Figure S3: S3A-D Different proportions of age, gender, stage, and grade were observed between the high and low-risk signature groups; **S3E-H** Risk signature score across ages, genders, stages, and grades.
