# Supplementary material for: Bioinformatics analysis and experimental validation of cuproptosis-related lncRNA LINC02154 in clear cell renal cell carcinoma
Source: BMC Cancer. 2023 Feb 16;23:160. doi: 10.1186/s12885-023-10639-2 (PMC9936708; doi:10.1186/s12885-023-10639-2)
Supplement: Supplementary file 2 — Supplementary Material 2 [file 12885_2023_10639_MOESM2_ESM.pdf]

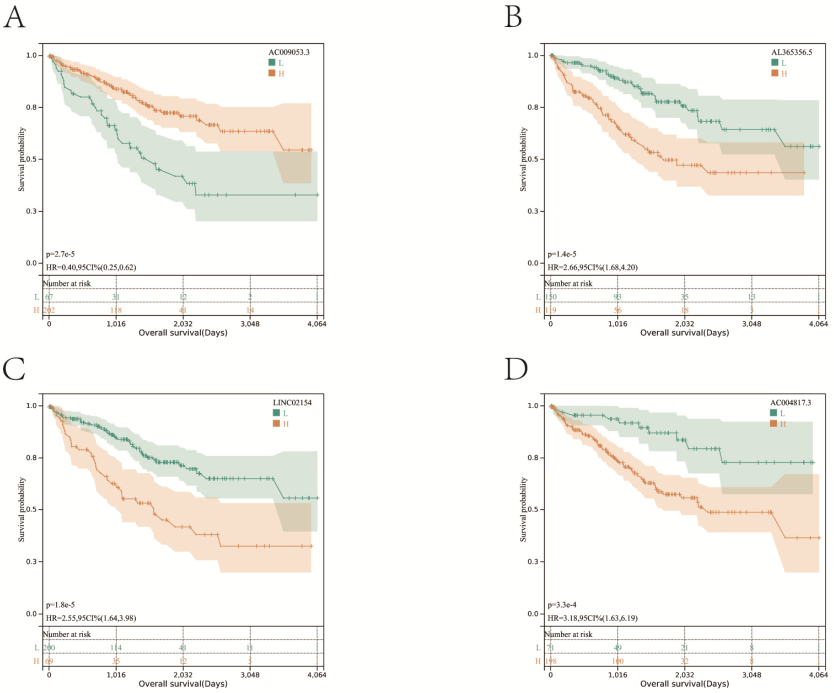

Supplementary Figure S2: S2A-D Single-gene survival analysis was performed on four genes in the risk signature in the testing set.
