# Supplementary material for: Bioinformatics analysis and experimental validation of cuproptosis-related lncRNA LINC02154 in clear cell renal cell carcinoma
Source: BMC Cancer. 2023 Feb 16;23:160. doi: 10.1186/s12885-023-10639-2 (PMC9936708; doi:10.1186/s12885-023-10639-2)
Supplement: Supplementary file 3 — Supplementary Material 3 [file 12885_2023_10639_MOESM3_ESM.pdf]

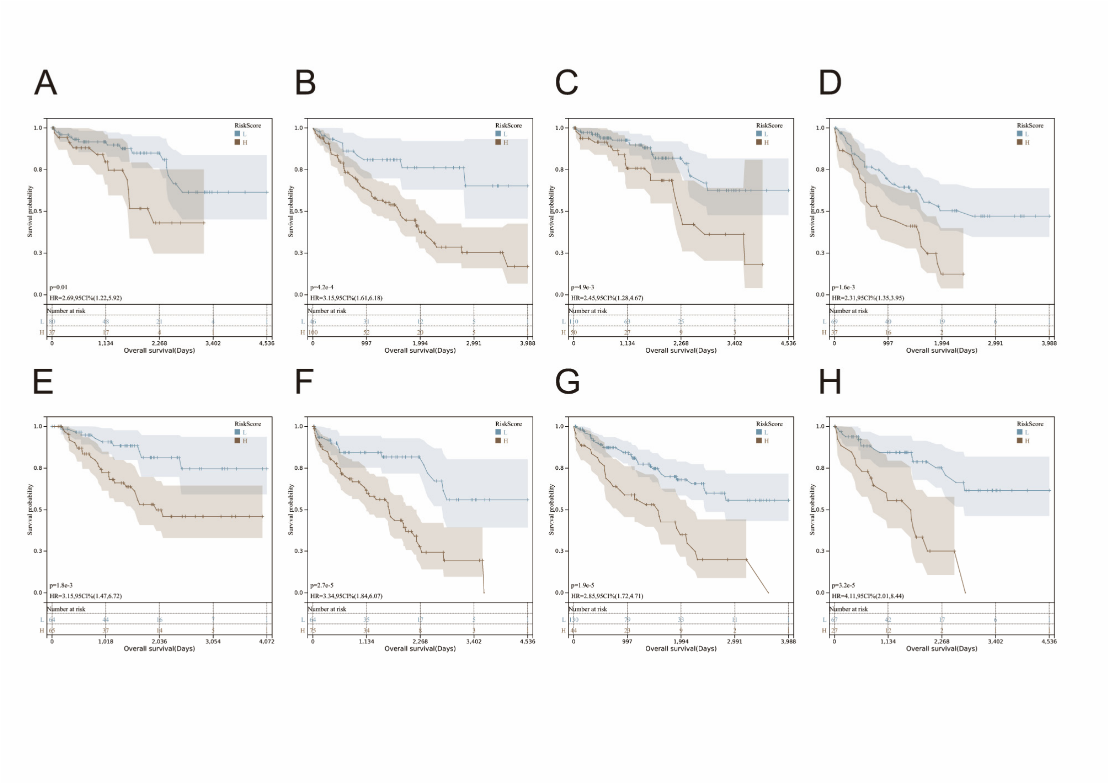

Supplementary Figure S4: S4A-H In the training set, grade, stage, age, and gender were significantly associated with outcomes.
