# Supplementary material for: Bioinformatics analysis and experimental validation of cuproptosis-related lncRNA LINC02154 in clear cell renal cell carcinoma
Source: BMC Cancer. 2023 Feb 16;23:160. doi: 10.1186/s12885-023-10639-2 (PMC9936708; doi:10.1186/s12885-023-10639-2)
Supplement: Supplementary file 4 — Supplementary Material 4 [file 12885_2023_10639_MOESM4_ESM.pdf]

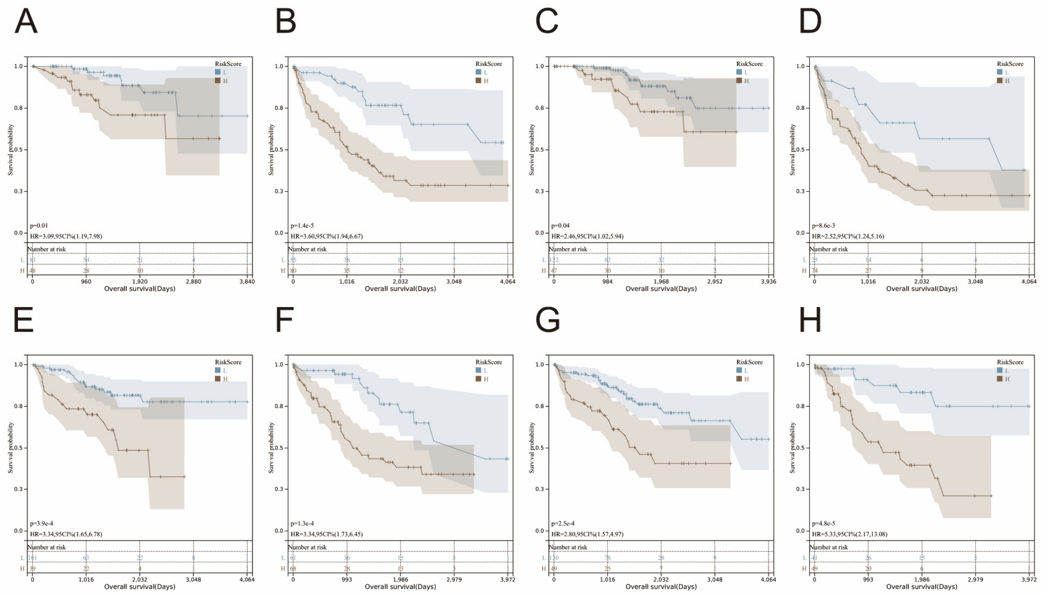

Supplementary Figure S5: S5A-H In the testing set, grade, stage, age, and gender were significantly associated with outcomes.
