# Supplementary material for: Bioinformatics analysis and experimental validation of cuproptosis-related lncRNA LINC02154 in clear cell renal cell carcinoma
Source: BMC Cancer. 2023 Feb 16;23:160. doi: 10.1186/s12885-023-10639-2 (PMC9936708; doi:10.1186/s12885-023-10639-2)
Supplement: Supplementary file 5 — Supplementary Material 5 [file 12885_2023_10639_MOESM5_ESM.pdf]

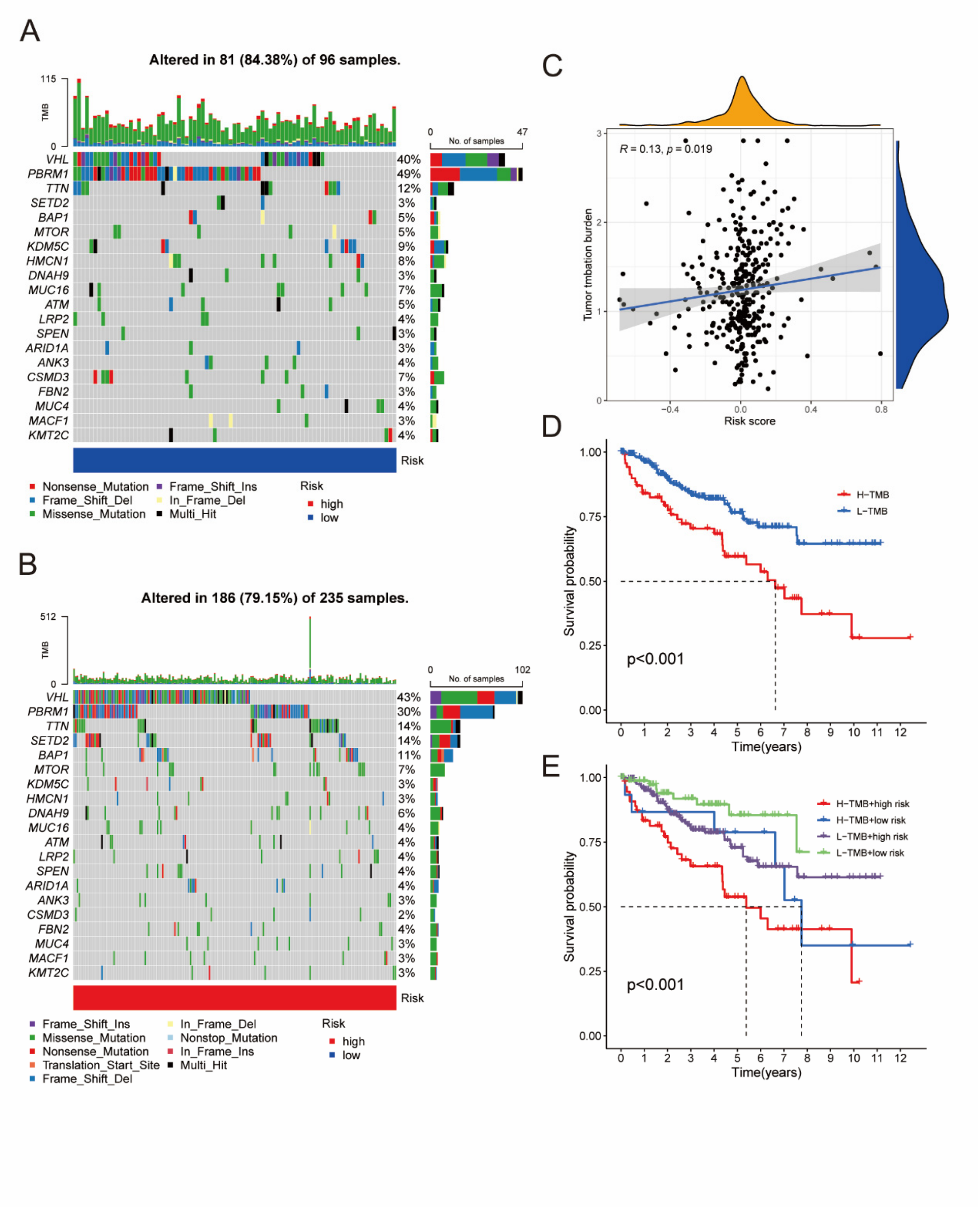

Supplementary Figure S6: S6A Tumor-associated gene mutation burden in samples from the low-risk group; **S6B** Tumor-associated gene mutation burden in samples from the high-risk group; **S6C** Positive relationship between risk score and tumor mutation burden; **S6D** Survival analysis of high and low tumor mutation burden groups; **S6E** Survival analysis of H−TMB + high-risk group, H−TMB + low-risk group, L−TMB +

high-risk group, L−TMB + low-risk group.
