# Supplementary material for: Bioinformatics analysis and experimental validation of cuproptosis-related lncRNA LINC02154 in clear cell renal cell carcinoma
Source: BMC Cancer. 2023 Feb 16;23:160. doi: 10.1186/s12885-023-10639-2 (PMC9936708; doi:10.1186/s12885-023-10639-2)
Supplement: Supplementary file 6 — Supplementary Material 6 [file 12885_2023_10639_MOESM6_ESM.pdf]

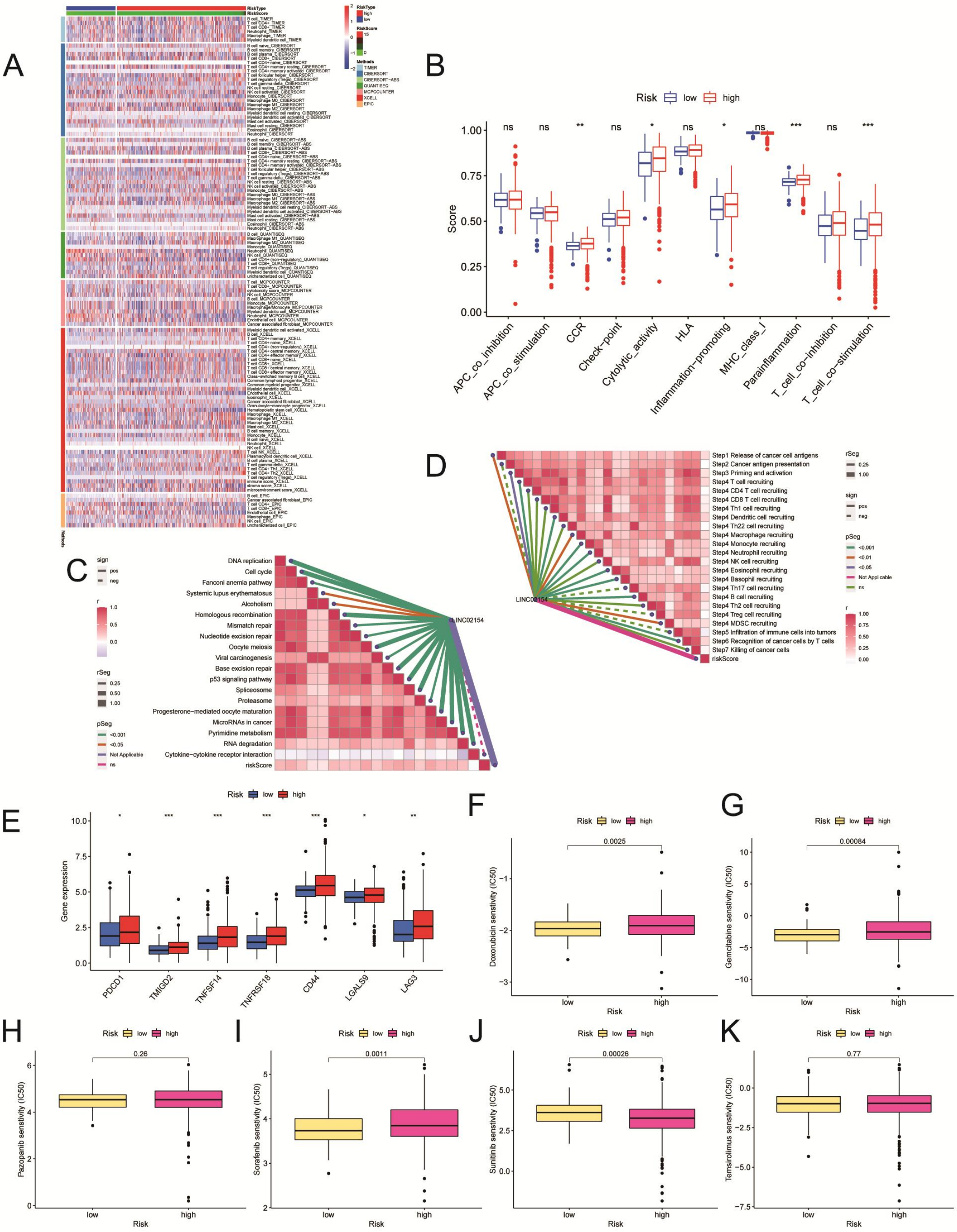

Supplementary Figure S7: S7A Relationship between risk score type and immune molecular typing; **S7B** Analysis of immune function in low- and high-risk groups; **S7C** Correlation analysis between risk score and positive markers related to immunotherapy; **S7D** Analysis of the correlation between risk scores and critical steps of the cancer immunity cycle; **S7E** Immune checkpoint analysis in high- and low-risk

groups; **S7F-L** Susceptibility analysis was performed separately for axitinib, doxorubicin, gemcitabine, pazopanib, sorafenib, sunitinib, and temsirolimus in high- and low-risk groups.
