# Supplementary figures and images for: Bioinformatics analysis and experimental validation of cuproptosis-related lncRNA LINC02154 in clear cell renal cell carcinoma
Source: BMC Cancer. 2023 Feb 16;23:160. doi: 10.1186/s12885-023-10639-2 (PMC9936708; doi:10.1186/s12885-023-10639-2)

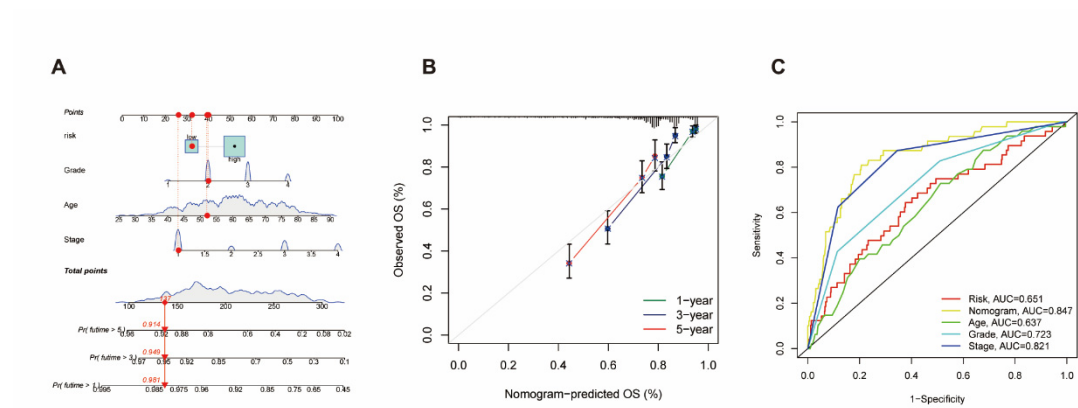

**Supplementary Figure S7: S7A Nomogram; S7B Calibration; S7C AUC.**

Supplement: Supplementary file 7 — Supplementary Material 7 [file 12885_2023_10639_MOESM7_ESM.pdf]
